# Current antimicrobial practice in febrile neutropenia across Europe and Asia: the EBMT Infectious Disease Working Party survey

**DOI:** 10.1038/s41409-020-0811-y

**Published:** 2020-02-11

**Authors:** Anke Verlinden, Malgorzata Mikulska, Nina Simone Knelange, Dina Averbuch, Jan Styczynski, on behalf of the Infectious Diseases Working Party (IDWP) of the European Group for Blood and Marrow Transplantation Group (EBMT)

**Affiliations:** 1 University Hospital Antwerp/University of Antwerp, Edegem, Belgium; 2 DISSAL, University of Genova and IRCCS Ospedale Policlinico San Martino, Genova, Italy; 3 EBMT Data Office, Leiden, Netherlands; 4 Hadassah-Hebrew University Medical Center, Jerusalem, Israel; 5 Department of Pediatric Hematology and Oncology, Collegium Medicum Nicolaus Copernicus University Torun, Bydgoszcz, Poland

**Keywords:** Infectious diseases, Haematological cancer

## Abstract

The aim of this survey was to summarize the current antimicrobial practice in febrile neutropenia and the presence of key aspects of antimicrobial stewardship. A questionnaire was sent to 567 centers, and complete responses were obtained from 194 (34.2%)*.* Fluoroquinolone and co-trimoxazole prophylaxis are used in 57.1% and 89.1%, respectively. In 66.4%, the first-line empirical therapy is piperacillin/tazobactam, whereas 10.9% use carbapenems. Empirical combination therapy is used in stable patients without history of resistant pathogens in 37.4%. De-escalation to monotherapy is performed within 3 days in 35.3% and after 10 days in 19.1%. Empirical addition of a glycopeptide is performed when fever persists more than 2–3 days in 60.8%. Empirical escalation to a broader spectrum agent is performed when fever persists more than 3–5 days in 71.4%. In case of positive blood cultures with a susceptible pathogen and uncomplicated presentation, 76.7% of centers de-escalate and 36.6% discontinue before neutrophil recovery. In fever of unknown origin with uncomplicated presentation, 54.1% of centers de-escalate and 49.5% discontinue before neutrophil recovery. Recommendations put forward in the ECIL guidelines are not widely implemented in clinical practice. Specific problems include overuse of carbapenems and combination therapy and unjustified addition of glycopeptides without further de-escalation or discontinuation.

## Introduction

Febrile neutropenia remains a challenging complication in hematopoietic stem cell transplant (HSCT) recipients. Although in more than half of cases an underlying infection cannot be identified, prompt administration of empiric broad-spectrum antibiotic therapy is standard of care as it decreases morbidity and mortality [1–3].

The worldwide presence of rising antimicrobial resistance has been confirmed in hematology patients and HSCT recipients [4, 5]. This led the European Conference on Infections in Leukemia (ECIL) to issue guidelines suggesting strategies of de-escalation and discontinuation of broad-spectrum antibiotic therapy under certain conditions [6]. The safety of de-escalation/discontinuation strategies prior to neutrophil recovery in high risk patients has been confirmed in several publications [7–9]. A recent critical appraisal on the use of fluoroquinolone prophylaxis (FP) concluded that the possible benefits on lowering the rate of bloodstream infections should be weighed against its impact in terms of toxicity and risk of increased colonization/infection with fluoroquinolone or multidrug resistant strains [10].

Implementation of the available guidelines is strongly affected by many factors, including local microbial epidemiology and resistance patterns, presence of rapid reporting of microbiological results, experience in management of infectious diseases, and historical antibiotic prescription habits [11]. The aim of this survey was to assess current general practice patterns with respect to antibiotic prophylaxis and empiric antibiotic treatment in febrile neutropenia, as well as the presence of key aspects of antimicrobial stewardship in EBMT centers.

## Methods

### The questionnaire

In August 2017, a total of 567 registered EBMT member centers in 57 countries were invited to complete the questionnaire and return it to the EBMT Data Center.

The questionnaire was designed to capture sufficient information to assess current practices in febrile neutropenia and gain an accurate impression on implementation of recent guidelines. It consisted of 54 questions (Supplementary Survey) and was divided into five sections, which included general information of the center, presence of key aspects of antimicrobial stewardship, policies on antibiotic prophylaxis, policies on empiric antibiotic therapy, and implementation of de-escalation/discontinuation strategies.

### Statistical analysis

Descriptive analysis was performed for all variables with absolute and percentage frequencies being reported. If applicable, percentage of missing values was reported and rates were calculated for number of valid responses as denominator.

A statistical comparison was performed to detect differences in the proportion of responses according to center characteristics such as performing autologous or allogeneic transplants, treating adults or children, geographical region and involvement of infectious diseases or microbiology departments (IDM) in writing guidelines and/or decision-making. Categorical variables were compared using chi-square or Fisher’s exact test if appropriate. Continuous variables were compared using Mann–Whitney test. A *p* value ≤ 0.05 was considered statistically significant.

## Results

A total of 194 (34.2%) centers from 40 (70%) countries returned the questionnaire*.* Geographically, 87 (44.8%) responses came from North-West (NW) Europe, 83 (42.8%) from South-East (SE) Europe, 21 (10.8%) from Asia, 2 (1.0%) from Africa (Algeria and Tunisia), and 1 (0.5%) from Australia. Due to small group size, centers from Africa and Australia were not included in geographical analysis. In 127 (65.5%) centers only adults are treated, in 35 (18.5%) only children, and in 32 (18.0%) both. In 40 (20.6%) centers only autologous HSCT are performed, in 3 (1.5%) only allogeneic HSCT, and in 151 (77.8%) both.

### Presence of key aspects of antimicrobial stewardship

Most centers [94.3% (182/193)] have written local guidelines on antibiotic policy in place, more often when performing both autologous and allogeneic HSCT versus autologous HSCT only [96.7% (145/150) versus 85.0% (35/40); *p* = 0.012] (Table 1/Supplementary Table 1). IDM are involved in writing guidelines in 61.0% (111/182)] and decision-making on antimicrobial treatment in 50.8% (98/193). IDM are more frequently involved in writing guidelines in SE and Asian versus NW centers [51.9% (41/79) and 44.4% (8/18) versus 25.6% (21/82); *p* = 0.004]. Surveillance cultures (nose and throat swab, urine and stool sample, central line insertion site swab) are performed in 82.9% (160/193), more frequently in SE and Asian versus NW centers [89.2% (74/83) and 90.0% (18/20) versus 74.7% (65/87); *p* = 0.030] and less frequently in centers performing only autologous HSCT versus both autologous and allogeneic HSCT [67.5% (27/40) versus 86.7% (130/150); *p* = 0.004]. Regular updates on local epidemiology and resistance patterns are received in 82.4% (159/193). Positive blood cultures are actively reported within 24 h in 94.3% (181/192). Rapid [98.9% (86/87) versus 91.5% (75/82) and 85.0% (17/20); *p* = 0.022] and active [98.9% (86/87) versus 89.0% (73/82) and 95.0% (19/20); *p* = 0.024] reporting is available more frequently in NW versus SE and Asian centers. The antimicrobial resistance profile is reported within 24 h after the result of a positive blood culture in 79.7% (153/192).

**Table 1:** Key aspects of antimicrobial stewardship and antibiotic policies on prophylaxis and empirical therapy.

*Key aspects of antimicrobial stewardship*	*n* = 194
Departmental guidelines on antibiotic policy are written	182/193 (94.3%)
By hematology department exclusively	71/182 (39.0%)
In cooperation with other service (infectiology/microbiology)	111/182 (61.0%)
Decisions on antimicrobial treatment are primarily made	
By hematology department exclusively	95/193 (49.2%)
In cooperation with other service (infectiology/microbiology)	98/193 (50.8%)
Performance of surveillance cultures	160/193 (82.9%)
Regular updates on (changes in) microbial epidemiology and resistance patterns	159/193 (82.4%)
Rapid (within 24 h) reporting of positive blood cultures	181/192 (94.3%)
Active (e.g., by telephone) reporting of positive blood cultures	181/192 (94.3%)
Resistance pattern of positive blood cultures reported within 24 h of culture becoming positive	153/192 (79.7%)
*Antibiotic policies on prophylaxis and empirical therapy*	
Fluoroquinolone prophylaxis being used	109/191 (57.1%)
Co-trimoxazole prophylaxis being used	172/191 (89.1%)
Combination therapy empirically in first line in stable patients without history of resistant pathogens	71/190 (37.4%)
Duration ≤ 3 days	24/68 (35.3%)
Duration ≥ 10 days	13/68 (19.1%)
First-line empiric antibiotic	
Piperacilline/tazobactam	117/189 (61.9%)
Fourth-generation cephalosporins	28/189 (14.8%)
Third-generation cephalosporins	18/189 (9.5%)
Carbapenems	20/189 (10.6%)
Other	6/189 (3.2%)
First-line empiric antibiotic—in monotherapy	
Piperacilline/tazobactam	79/119 (66.4%)
Fourth-generation cephalosporins	17/119 (14.3%)
Third-generation cephalosporins	7/119 (5.9%)
Carbapenems	13/119 (10.9%)
Other	3/119 (2.5%)
First-line empiric antibiotic—as part of combination therapy	
Piperacilline/tazobactam	38/70 (54.3%)
Fourth-generation cephalosporins	11/70 (15.7%)
Third-generation cephalosporins	11/70 (15.7%)
Carbapenems	7/70 (10.0%)
Other	3/70 (4.3%)
Association of a glycopeptide empirically in case of persistent fever	115/189 (60.8%)
Escalation to a broader spectrum antibiotic empirically in case of persistent fever	135/189 (71.4%)

This table summarizes all valid responses from centers on questions concerning key aspects of antimicrobial stewardship and antibiotic policies on prophylaxis and empirical therapy.

### Antibiotic policies: prophylaxis and empirical therapy

Antibacterial prophylaxis with FP is used in 57.1% (109/191), significantly more frequently in SE and Asian versus NW centers [71.6% (58/81) and 60.0% (12/20) versus 43.7% (38/87); *p* = 0.001)] and in centers treating adults versus children [62.9% (78/124) versus 28.6% (10/35); *p* < 0.001] (Table 1/Supplementary Table 1). Ciprofloxacin is the most commonly used agent both in autologous [67.0% (65/97)] and allogeneic [63.3% (75/90)] HSCT, followed by levofloxacin [27.8% (27/97) in autologous and 32.2% (29/90) in allogeneic]. FP is initiated either at onset of conditioning [57.3% (63/110)] or at onset of neutropenia [31.8% (35/110)] and continued until recovery of neutrophils [83.5% (91/109)] or discharge [10.1% (11/109)].

Antipneumocystis prophylaxis with co-trimoxazole is used in 89.1% (172/191), significantly more frequently in centers performing both autologous and allogeneic HSCT versus only autologous HSCT [91.9% (137/149) versus 77.5% (31/40); *p* = 0.020]. Co-trimoxazole prophylaxis is initiated either after engraftment [58.1% (100/172)] or at onset of conditioning [25.0% (43/172)]. The duration of co-trimoxazole prophylaxis varies considerably, with some centers using a time-dependent stop date (6 or 12 months post allogeneic HSCT) and others based on immunosuppressive treatment and/or immunological recovery.

A total of 62.6% (119/190) use monotherapy empirically in first line. In these centers, piperacillin/tazobactam is preferred in 66.4% (79/119), fourth-generation cephalosporins in 14.3% (17/119), carbapenems in 10.9% (13/119), third-generation cephalosporins in 5.9% (7/119), and no clear preference in 2.5% (3/119). Whereas piperacillin/tazobactam is used most frequently in all regions, the second most frequently used are third- and fourth-generation cephalosporins in NW centers [both 9.1% (5/55)], fourth-generation cephalosporins in SE centers [22.0% (11/50)] and carbapenems in Asian centers [15.4% (2/13)]. Piperacillin/tazobactam is used more frequently as first-line empirical monotherapy in NW versus SE centers [74.5% (41/55) versus 56.0% (28/50); *p* = 0.046] and in centers treating adults versus children [73.8% (59/80) versus 47.1% (8/17); *p* = 0.031].

Empirical combination therapy (mainly with aminoglycosides) is used as first line in 37.4% (71/190), with a tendency towards more frequent use in centers treating children versus adults [51.4% (18/35) versus 36.0% (45/125); *p* = 0.099]. The use of empirical combination therapy does not differ significantly between centers performing only autologous transplants [37.5% (15/40)] or both autologous and allogeneic [38.1% (56/147)]. In these centers, piperacillin/tazobactam is the main component in 54.3% (38/70), third- and fourth-generation cephalosporins equally in 15.7% (11/70), carbapenems in 10.0% (7/70), and no clear preference in 4.3% (3/70). Whereas piperacilline/tazobactam is used most frequently in all regions, the second most frequently used are third-generation cephalosporins in NW centers [31.0% (9/29)], fourth-generation cephalosporins in SE centers [25.0% (8/32)], and carbapenems in Asian centers [28.6% (2/7)]. Third-generation cephalosporins are used more frequently as main component of first-line empirical combination therapy in NW versus SE centers [31.0% (9/29) versus 6.3% (2/32); *p* = 0.012] and in centers performing autologous HSCT versus both autologous and allogeneic HSCT [33.3% (5/15) versus 10.9% (6/55); *p* = 0.049].

Empirical addition of a glycopeptide when fever persists for more than 2–3 days is performed in 60.8% (115/189), more frequently in SE and Asian versus NW centers [71.6% (58/81) and 65.0% (13/20) versus 51.8% (44/85); *p* = 0.030] and in centers using FP [67.9% (72/106) versus 51.2% (42/82); *p* = 0.020].

Empirical escalation to a broader spectrum agent is performed when fever persists for more than 3–5 days in 71.4% (135/189), more frequently in SE and Asian versus NW centers [82.9% (68/82) and 80.0% (16/20) versus 60.7% (51/84); *p* = 0.004] and in centers using FP [83.2% (89/107) versus 55.6% (45/81); *p* < 0.001]. Centers where IDM are involved in writing guidelines perform less empirical escalation to a broader spectrum agent [63.0% (68/108) versus 82.6% (57/69); *p* = 0.005].

### Implementation of de-escalation/discontinuation strategies

In 35.3% (24/68) of centers using empirical combination therapy de-escalation to monotherapy is performed within 3 days, whereas in 19.1% (13/68) combination therapy is continued for more than 10 days (Table 2/Supplementary Tables 1 and 2). NW centers are more likely to de-escalate to monotherapy within 3 days than SE centers [50.0% (14/28) versus 20.0% (6/30); *p* = 0.016] and SE centers continue combination therapy more frequently for more than 10 days [30.0% (9/30) versus 7.1% (2/28); *p* = 0.026]. When IDM are involved in writing guidelines and decision-making, centers tend to de-escalate more frequently to monotherapy within 3 days [respectively 44.4% (16/36) versus 24.1% (7/29); *p* = 0.089 and 45.1% (14/31) versus 27.0% (10/37); *p* = 0.119].

**Table 2:** Implementation of de-escalation/discontinuation strategies.

*Empirical de-escalation of combination therapy*	*n* = 194
Combination therapy empirically in first line in stable patients without history of resistant pathogens	71/190 (37.4%)
Duration ≤ 3 days	24/68 (35.3%)
Duration ≥ 10 days	13/68 (19.1%)
*De-escalation of antibiotics in specific situations*	
Positive blood culture with susceptible pathogen with uncomplicated presentation	143/186 (76.8%)
Positive blood culture with susceptible pathogen with severe presentation, improved on empirical therapy	82/183 (44.8%)
Clinically documented infection with uncomplicated presentation, afebrile on empirical therapy	113/183 (61.7%)
Clinically documented infection with severe presentation, improved and afebrile on empirical therapy	71/184 (38.6%)
Fever of unknown origin with uncomplicated presentation, afebrile on empirical therapy	100/185 (54.1%)
Fever of unknown origin with severe presentation, improved and afebrile on empirical therapy	56/185 (30.3%)
*Stop before neutrophil recovery in specific situations*	
Positive blood culture with susceptible pathogen with uncomplicated presentation	68/186 (36.6%)
Positive blood culture with susceptible pathogen with severe presentation, improved on empirical therapy	37/186 (19.9%)
Clinically documented infection with uncomplicated presentation, afebrile on empirical therapy	76/186 (40.9%)
Clinically documented infection with severe presentation, improved and afebrile on empirical therapy	39/185 (21.1%)
Fever of unknown origin with uncomplicated presentation, afebrile on empirical therapy	91/184 (49.5%)
Fever of unknown origin with severe presentation, improved and afebrile on empirical therapy	40/184 (21.7%)
Probable/proven pulmonary aspergillosis with uncomplicated presentation, afebrile on antifungal therapy	41/183 (22.4%)
Probable/proven pulmonary aspergillosis with severe presentation, improved and afebrile on antifungal therapy	33/183 (18.0%)
*How long is antibiotic therapy generally continued*	
Positive blood culture	
<7 days	2/182 (1.1%)
7–10 days	51/182 (28.0%)
11–14 days	63/182 (34.6%)
15–21 days	11/182 (6.0%)
Until end of neutropenia	55/182 (30.2%)
Clinically documented infection	
<7 days	4/182 (2.2%)
7–10 days	57/182 (31.3%)
11–14 days	63/182 (34.6%)
15–21 days	7/182 (3.8%)
Until end of neutropenia	51/182 (28.0%)
Fever of unknown origin	
<7 days	32/182 (17.6%)
7–10 days	60/182 (33.0%)
11–14 days	27/182 (14.8%)
15–21 days	3/182 (1.6%)
Until end of neutropenia	60/182 (33.0%)

This table summarizes all valid responses from centers on questions concerning Implementation of de-escalation/discontinuation strategies.

In case of positive blood cultures with a susceptible pathogen and uncomplicated presentation, 76.7% (143/186) de-escalate the empirically started antibiotic regimen. In the same situation, discontinuation of antibiotics prior to neutrophil recovery is performed in 36.6% (68/186), more frequently in NW versus SE and Asian centers [47.6% (39/82) versus 28.0% (23/82) and 31.6% (6/19); *p* = 0.031] and in centers where IDM are involved in writing guidelines [44.9% (48/107) versus 26.9% (18/67); *p* = 0.017]. Antibiotics are generally discontinued within 7 days. Centers that empirically escalate to a broader spectrum agent in case of persistent fever discontinue less frequently in this clinical scenario [30.8% (41/133) versus 51.9% (27/52); *p* = 0.007].

In case of positive blood cultures with a susceptible pathogen and severe presentation which improved on empirical therapy, 44.8% (82/183) de-escalate the empirically started antibiotic regimen, more frequently in centers where IDM are involved in decision-making [53.2% (50/94) versus 36.4% (32/88); *p* = 0.023]. In the same situation discontinuation of antibiotics prior to neutrophil recovery is performed in 19.9% (37/186), more frequently in NW than SE centers [26.8% (22/82) versus 14.6% (12/82); *p* = 0.054] and in centers where IDM are involved in writing guidelines [27.1% (29/107) versus 11.9% (8/67); *p* = 0.017].

When confronted with a clinically documented infection with uncomplicated presentation, 61.7% (113/183) de-escalate the empirically started antibiotic regimen. In the same situation discontinuation of antibiotics prior to neutrophil recovery is performed in 40.9% (76/186), more frequently in NW versus SE and Asian centers [59.8% (49/82) versus 28.0% (23/82) and 21.1% (4/19); *p* < 0.001] and in centers where IDM are involved in writing guidelines [48.6% (52/107) versus 32.8% (22/67); *p* = 0.041].

When confronted with a clinically documented infection with severe presentation which improved on empirical therapy, 38.6% (71/184) de-escalate the empirically started antibiotic regimen, more likely in centers where IDM are involved in decision-making [45.2% (42/93) versus 32.2% (29/90); *p* = 0.073]. In the same situation discontinuation of antibiotics prior to neutrophil recovery is performed in 21.1% (39/185).

In case of fever of unknown origin (FUO) with uncomplicated presentation, 54.1% (100/185) de-escalate the empirically started antibiotic regimen, more frequently in centers treating children than adults [71.4% (25/35) versus 52.5% (63/120), *p* = 0.047] and in centers using empirical combination therapy [64.7% (44/68) versus 47.9% (56/117); *p* = 0.027]. In the same situation discontinuation of antibiotics prior to neutrophil recovery is performed in 49.5% (91/184), more frequently in NW and Asian versus SE centers [62.5% (50/80) and 57.9% (11/19) versus 36.6% (30/82); *p* = 0.003], in centers that perform only autologous HSCT versus both autologous/allogeneic HSCT [62.5% (25/40) versus 46.1% (65/141); *p* = 0.067] and in centers where IDM are involved in decision-making [56.4% (53/94) versus 42.7% (38/89); *p* = 0.064]. Antibiotics are generally discontinued after 2–7 days without fever. Centers that empirically associate a glycopeptide and empirically escalate to a broader spectrum agent in case of persistent fever, discontinue antibiotics less frequently in case of uncomplicated FUO [respectively 43.2% (48/111) versus 59.7% (43/72); *p* = 0.029 and 45.8% (60/131) versus 59.6% (31/52); *p* = 0.092].

In case of FUO with severe presentation which improved on empirical therapy, 30.3% (56/185) de-escalate the empirically started antibiotic regimen, more frequently in centers treating children versus adults [48.6% (17/35) versus 27.5% (33/120), *p* = 0.019] and centers where IDM are involved in decision-making [38.3% (36/94) versus 22.2% (20/90); *p* = 0.018]. In this same situation discontinuation of antibiotics prior to neutrophil recovery is performed in 21.7% (40/184), more frequently in centers where IDM are involved in writing guidelines [30.2% (32/106) versus 12.1% (8/66); *p* = 0.006].

When confronted with patients with probable/proven pulmonary aspergillosis, either with uncomplicated or severe presentation, who became afebrile on antifungal therapy, centers discontinue antibiotics prior to neutrophil recovery in 22.4% (41/183) and 18.0% (33/183), respectively.

Whereas most centers generally continue antibiotic therapy for 7–14 days, a third of centers continue treatment until the end of neutropenia in any given scenario. In centers where IDM are involved in decision-making, antibiotics are more frequently discontinued before neutrophil recovery in case of positive blood cultures [37.9% (33/87) versus 23.4% (22/94); *p* = 0.034], clinically documented infection [35.6% (31/87) versus 21.3% (20/94); *p* = 0.032] and FUO [41.4% (36/87) versus 25.5% (24/94); *p* = 0.024]. There were no significant differences in treatment duration in relation to the center’s policy on the use of FP, combination therapy, or carbapenems in first line, empirical association of a glycopeptide or empirical escalation to a broader spectrum agent.

## Discussion

The management of febrile neutropenia in HSCT recipients remains challenging as there are many possible infectious and noninfectious causes for fever in these patients. In view of rising antimicrobial resistance in hematology patients, ECIL issued guidelines in 2011 introducing the concept of escalation/de-escalation of empirical therapy and suggesting discontinuation of broad-spectrum antibiotic therapy under certain conditions, which has been confirmed safe in several recent publications [4–9]. Compliance with these recommendations has never been assessed and this survey was performed to quantify their current implementation rate in EBMT centers. In the survey there were no specifications made about circumstances or timing in the transplant journey, but typically questions on febrile neutropenia involve mainly pre-engraftment period. The rate of response to this questionnaire was similar to prior surveys [12]. It demonstrated important discrepancies between guidelines and practices that should be addressed in order to optimize antimicrobial therapy and diminish development of resistance. Local definitions for fever, neutrophil cutoff and interpretation of clinical status of the patient (stable versus severe presentation) were used. This might cause some limitations to the interpretation of the data, but on the other hand represents the real world situation where definitions and practices might differ between centers and even between single clinicians within a center.

ECIL guidelines recommend an escalation approach, meaning monotherapy with noncarbapenem beta-lactams in stable patients without history of colonization/infection with resistant bacteria. Still, a third of centers use empirical combination therapy in first line and 10% use carbapenems. This finding does not correlate with geographical location and cannot be explained solely by epidemiological presence of multidrug resistant bacteria. Centers using empirical combination therapy and/or carbapenems often did not define themselves as using a de-escalation approach, indicating the need for education on escalation/de-escalation principles.

Streamlining of initial combination therapy is recommended after 72 h, including discontinuation of combination therapy if resistant bacteria were not cultured. However, only a third of centers using combination therapy, de-escalate within 3 days and SE centers are more likely to extend combination therapy for more than 10 days. Although timing of de-escalation depends on speed of antimicrobial susceptibility testing, the large majority of centers (79.7% of total and 84.1% of SE centers) noted swift reporting of resistance figures. In case of positive blood cultures with a susceptible pathogen, many centers do not de-escalate, indicating clinicians may not feel confident enough in results of susceptibility testing and/or fear a decline after de-escalation.

ECIL guidelines emphasize that there is no need to escalate treatment in stable patients with persisting fever. This recommendation is supported by previous clinical trials which noted that the median time to defervescence in high risk neutropenic patients is 5 days [13]. Nevertheless, more than half of centers add a glycopeptide (60.8%) and/or escalate to a broader spectrum agent (71.4%) empirically and the minority of these centers de-escalate later on. These empirical additions/escalations are performed significantly more frequently in centers from SE Europe and Asia, centers using FP and centers without IDM involvement.

The duration of empirical treatment is a debatable issue. IDSA and ESMO guidelines recommend continuing antibiotic therapy until neutrophil recovery [14, 15]. ECIL guidelines recommend considering treatment discontinuation after 72 h or later in hemodynamically stable patients with FUO who are afebrile for at least 48 h, irrespective of neutrophil count or expected duration of neutropenia [6]. However, results of this survey show that in case of FUO, discontinuation of antibiotic therapy within 7 days is only performed in 17.6% and one-third of centers continue treatment until neutrophil recovery. Centers from SE Europe and Asia are less likely to discontinue antibiotic therapy before neutrophil recovery.

Although FP is currently recommended by several guidelines, the benefits should be weighed against the risks [10]. Our survey demonstrated that half of centers provide FP, significantly more frequently in centers treating adults and in SE Europe and Asia. Reduced value of FP is expected in the presence of the higher resistance rates reported in the southern regions and one would expect changes in practices accordingly. However, the results of our survey show the opposite, which can be due to historically higher rates of FP use in these regions. Centers using FP are more likely to empirically add a glycopeptide and/or escalate to a broader spectrum agent in stable patients with persistent fever. Nonetheless these centers are not more likely to de-escalate or discontinue the empirical antibiotic treatment.

Centers from SE Europe more frequently use FP, empirically add a glycopeptide and/or escalate to a broader spectrum agent in stable patients with persistent fever and continue combination therapy for a longer period of time. On the other hand they are less likely to de-escalate or stop antibiotic therapy prior to neutrophil recovery. These practices may result from higher resistance rates [4, 5], but can also be the cause of these resistance rates. Nonetheless, they create a vicious circle of higher antibiotic consumption and higher resistance rates, which can only be broken by implementing antimicrobial stewardship.

Most key aspects of antimicrobial stewardship are available in a great majority of centers, although only around half are advised by IDM when writing guidelines or taking decisions on antimicrobial treatment. This is an important finding, as empirical escalation to a broader spectrum agent in stable patients with persistent fever was performed less frequently and de-escalation/discontinuation rates were significantly higher in centers with IDM involvement. This emphasizes the importance of collaboration between the hematology departments and their IDM counterparts to improve antimicrobial stewardship as a first step towards lowering resistance rates.

## Conclusion

Recommendations put forward in the ECIL guidelines are not widely implemented in clinical practice throughout EBMT centers, less frequently in SE Europe and Asia, which may correlate with higher resistance rates. Specific problems include overuse of combination therapy and carbapenems and unnecessary addition of glycopeptides without further de-escalation. Despite growing evidence on the safety of treatment discontinuation, a large proportion of centers continue antibiotic therapy until neutrophil recovery. Education on escalation and de-escalation practices, dissemination of the guidelines and available evidence can lead to a change in practice, which is important to diminish growing resistance rates. This should be accompanied by continuous monitoring of resistance patterns and clinical patient outcome data.

## Supplementary information

Supplementary Table 1: Comparison by geographical region, type of transplant and patient age

Supplementary Table 2: Implementation of de-escalation/discontinuation strategies relative to other variables

Survey
