# Supplementary material for: Current antimicrobial practice in febrile neutropenia across Europe and Asia: the EBMT Infectious Disease Working Party survey
Source: Bone Marrow Transplant. 2020 Feb 11;55(8):1588–94. doi: 10.1038/s41409-020-0811-y (PMC7391284; doi:10.1038/s41409-020-0811-y)
Supplement: Supplementary file 1 — Supplementary Table 1: Comparison by geographical region, type of transplant and patient age [file 41409_2020_811_MOESM1_ESM.docx]

|  |  |  |  |  |  |  |  |  |  |
| --- | --- | --- | --- | --- | --- | --- | --- | --- | --- |
| Key aspects of antimicrobial stewardship | NW | SE | Asia | Adults | Children | Both | Auto | Allo | Both |
|  | n = 87 | n = 83 | n = 21 | n = 127 | n = 35 | n = 32 | n = 40 | n = 3 | n = 151 |
|  |  |  |  |  |  |  |  |  |  |
| Departmental guidelines on antibiotic policy are written | 82/86 (95.3%) | 79/83 (95.2%) | 18/21 (85.7%) | 117/126 (92.9%) | 34/35 (97.1%) | 31/32 (96.9%) | **35/40 (85.0%)** | **3/3 (100%)** | **145/150 (96.7%)** |
| By hematology department exclusively | **61/82 (74.4%)** | **38/79 (48.1%)** | **10/18 (55.6%)** | 59/117 (42.7%) | 12/34 (35.3%) | 9/31 (29.0%) | 9/34 (26.5%) | 1/3 (33.3%) | 61/145 (42.1%) |
| In cooperation with other service (infectious diseases/microbiology) | **21/82 (25.6%)** | **41/79 (51.9%)** | **08/18 (44.4%)** | 67/117 (57.3%) | 22/34 (64.7%) | 22/31 (71.0%) | 25/34 (73.5%) | 2/3 (66.7%) | 85/145 (57.9%) |
| Decisions on antimicrobial treatment are primarily made |  |  |  |  |  |  |  |  |  |
| By hematology department exclusively | 38/87 (43.7%) | 47/82 (57.3%) | 08/21 (38.1%) | 59/126 (46.8%) | 20/35 (57.1%) | 16/32 (50.0%) | 15/40 (37.5%) | 2/3 (66.7%) | 78/150 (52.0%) |
| In cooperation with other service (infectious diseases/microbiology) | 49/87 (56.3%) | 35/82 (42.7%) | 13/21 (61.9%) | 67/126 (53.2%) | 15/35 (42.9%) | 16/32 (50.0%) | 25/40 (62.5%) | 1/3 (33.3%) | 72/150 (48.0%) |
| Performance of surveillance cultures | **65/87 (74.7%)** | **74/83 (89.2%)** | **18/20 (90.0%)** | 105/126 (83.3%) | 31/35 (88.6%) | 24/32 (75.0%) | **27/40 (67.5%)** | **3/3 (100%)** | **130/150 (86.7%)** |
| Regular updates on (changes in) microbial epidemiology and resistance patterns | 70/87 (80.5%) | 73/83 (88.0%) | 14/20 (70.0%) | 106/126 (84.1%) | 27/35 (77.1%) | 26/32 (81.3%) | 33/40 (82.5%) | 2/3 (66.7%) | 124/150 (82.7%) |
| Rapid (within 24 hours) reporting of positive blood cultures | **86/87 (98.9%)** | **75/82 (91.5%)** | **17/20 (85.0%)** | 116/125 (92.8%) | 35/35 (100%) | 30/32 (93.8%) | 39/40 (97.5%) | 3/3 (100%) | 139/149 (93.3%) |
| Active (e.g. by telephone) reporting of positive blood cultures | **86/87 (98.9%)** | **73/82 (89.0%)** | **19/20 (95.0%)** | 115/125 (92.0%) | 35/35 (100%) | 31/32 (96.9%) | 38/40 (95.0%) | 3/3 (100%) | 140/149 (94.0%) |
| Resistance pattern of positive blood cultures reported within 24 hours of culture becoming positive | 68/87 (78.2%) | 69/82 (84.1%) | 14/20 (70.0%) | 100/125 (80.0%) | 27/35 (77.1%) | 26/32 (81.3%) | 33/40 (82.5%) | 3/3 (100%) | 117/149 (78.5%) |
|  |  |  |  |  |  |  |  |  |  |
| Antibiotic policies on prophylaxis and empirical therapy | NW | SE | Asia | Adults | Children | Both | Auto | Allo | Both |
|  | n = 87 | n = 83 | n = 21 | n = 127 | n = 35 | n = 32 | n = 40 | n = 3 | n = 151 |
|  |  |  |  |  |  |  |  |  |  |
| Fluoroquinolone prophylaxis being used | **38/87 (43.7%)** | **58/81 (71.6%)** | **12/20 (60.0%)** | **78/124 (62.9%)** | **10/35 (28.6%)** | **21/32 (65.6%)** | 19/40 (47.5%) | 1/3 (33.3%) | 89/148 (60.1%) |
| Cotrimoxazole prophylaxis being used | 76/87 (87.4%) | 76/82 (92.7%) | 18/20 (90.0%) | 108/125 (86.4%) | 34/35 (97.1%) | 29/32 (90.6%) | **31/40 (77.5%)** | **3/3 (100%)** | **137/149 (91.9%)** |
|  |  |  |  |  |  |  |  |  |  |
| Combination therapy empirically in first line in stable patients without history of resistant pathogens | 30/85 (35.3%) | 32/82 (39.0%) | 7/20 (35.0%) | 45/125 (36.0%) | 18/35 (51.4%) | 8/30 (26.7%) | 15/40 (37.5%) | 0/3 (0%) | 56/147 (38.1%) |
| Duration =< 3 days | **14/28 (50.0%)** | **6/30 (20.0%)** | **3/8 (37.5%)** | 14/43 (32.6%) | 7/18 (38.9%) | 3/7 (42.9%) | 4/13 (30.8%) |  | 20/55 (36.4%) |
| Duration >= 10 days | **2/28 (7.1%)** | **9/30 (30.0%)** | **2/8 (25.0%)** | 9/43 (20.9%) | 3/18 (16.7%) | 1/7 (14.3%) | 2/13 (15.4%) |  | 11/55 (20.0%) |
| First line empiric antibiotic |  |  |  |  |  |  |  |  |  |
| Piperacillin/tazobactam | 55/84 (65.5%) | 45/82 (54.9%) | 14/20 (70.0%) | 80/125 (68.0%) | 20/34 (58.8%) | 17/30 (56.7%) | 27/40 (67.5%) | 2/3 (66.7%) | 88/146 (60.3%) |
| 4th generation cephalosporins | **8/84 (9.5%)** | **19/82 (23.2%)** | **1/20 (5.0%)** | 14/125 (11.2%) | 8/34 (23.5%) | 6/30 (20.0%) | 5/40 (12.5%) | 1/3 (33.3%) | 22/146 (15.1%) |
| 3rd generation cephalosporins | **13/84 (15.5%)** | **3/82 (3.7%)** | **2/20 (10.0%)** | 12/125 (9.6%) | 2/34 (5.9%) | 4/30 (13.3%) | 6/40 (15.0%) | 0/3 (0%) | 12/146 (8.2%) |
| Carbapenems | 7/84 (8.3%) | 10/82 (12.2%) | 3/20 (15.0%) | 14/125 (11.2%) | 3/34 (8.8%) | 3/30 (10.0%) | 1/40 (2.5%) | 0/3 (0%) | 19/146 (13.0%) |
| Other | 1/84 (1.2%) | 5/82 (6.1%) | 0/20 (0.0%) | 5/125 (4.0%) | 1/34 (2.9%) | 0/30 (0.0%) | 1/40 (2.5%) | 0/3 (0%) | 5/146 (3.4%) |
| First line empiric antibiotic - in monotherapy |  |  |  |  |  |  |  |  |  |
| Piperacillin/tazobactam | **41/55 (74.5%)** | **28/50 (56.0%)** | **9/13 (69.2%)** | **59/80 (73.8%)** | **8/17 (47.1%)** | **12/22 (54.5%)** | 19/25 (76.0%) | 2/3 (66.7%) | 58/91 (63.7%) |
| 4th generation cephalosporins | 5/55 (9.1%) | 11/50 (22.0%) | 1/13 (7.7%) | **6/80 (7.5%)** | **5/17 (29.4%)** | **6/22 (27.3%)** | 3/25 (12.0%) | 1/3 (33.3%) | 13/91 (14.3%) |
| 3rd generation cephalosporins | 4/55 (7.3%) | 1/50 (2.0%) | 2/13 (15.4%) | 4/80 (5.0%) | 1/17 (5.9%) | 2/22 (9.3%) | 1/25 (4.0%) | 0/3 (0%) | 6/91 (6.6%) |
| Carbapenems | 5/55 (9.1%) | 7/50 (14.0%) | 1/13 (7.7%) | 9/80 (11.3%) | 2/17 (11.8%) | 2/22 (9.3%) | 1/25 (4.0%) | 0/3 (0%) | 12/91 (13.2%) |
| Other | 0/55 (0%) | 3/50 (6.0%) | 0/13 (0%) | 2/80 (2.5%) | 1/17 (5.9%) | 0/22 (0%) | 1/25 (4.0%) | 0/3 (0%) | 2/91 (2.2%) |
| First line empiric antibiotic - as part of combination therapy |  |  |  |  |  |  |  |  |  |
| Piperacillin/tazobactam | 14/29 (48.3%) | 17/32 (53.1%) | 5/7 (71.4%) | 21/45 (46.7%) | 12/17 (70.6%) | 5/8 (62.5%) | 8/15 (53.3%) |  | 30/55 (54.5%) |
| 4th generation cephalosporins | 3/19 (10.3%) | 8/32 (25.0%) | 0/7 (0%) | 8/45 (17.8%) | 3/17 (17.6%) | 0/8 (0%) | 2/15 (13.3%) |  | 9/55 (16.4%) |
| 3rd generation cephalosporins | **9/29 (31.0%)** | **2/32 (6.3%)** | **0/7 (0%)** | 8/45 (17.8%) | 1/17 (5.9%) | 2/8 (25.0%) | **5/15 (33.3%)** |  | **6/55 (10.9%)** |
| Carbapenems | 2/29 (6.9%) | 3/32 (9.4%) | 2/7 (28.6%) | 5/45 (11.1%) | 1/17 (5.9%) | 1/8 (12.5%) | 0/15 (0%) |  | 7/55 (12.7%) |
| Other | 1/29 (3.4%) | 2/32 (6.3%) | 0/7 (0%) | 3/45 (6.7%) | 0/17 (0%) | 0/8 (0%) | 0/15 (0%) |  | 3/55 (5.5%) |
|  |  |  |  |  |  |  |  |  |  |
| Association of a glycopeptide empirically in case of persistent fever | **44/85 (51.8%)** | **58/81 (71.6%)** | **13/20 (65.0%)** | 73/124 (58.9%) | 24/35 (68.6%) | 18/30 (60.0%) | 22/40 (55.0%) | 1/3 (33.3%) | 92/146 (63.0%) |
| Escalation to a broader spectrum antibiotic empirically in case of persistent fever | **51/84 (60.7%)** | **68/82 (82.9%)** | **16/20 (80.0%)** | 89/124 (71.8%) | 23/35 (65.7%) | 23/30 (76.7%) | 31/40 (77.5%) | 0/3 (0%) | 104/146 (71.2%) |
|  |  |  |  |  |  |  |  |  |  |
| Implementation of de-escalation/discontinuation strategies |  |  |  |  |  |  |  |  |  |
|  | NW | SE | Asia | Adults | Children | Both | Auto | Allo | Both |
| Empirical de-escalation of combination therapy | n = 87 | n = 83 | n = 21 | n = 127 | n = 35 | n = 32 | n = 40 | n = 3 | n = 151 |
|  |  |  |  |  |  |  |  |  |  |
| Combination therapy empirically in first line in stable patients without history of resistant pathogens | 30/85 (35.3%) | 32/82 (39.0%) | 7/20 (35.0%) | 45/125 (36.0%) | 18/35 (51.4%) | 8/30 (26.7%) | 15/40 (37.5%) | 0/3 (0%) | 56/147 (38.1%) |
| Duration =< 3 days | **14/28 (50.0%)** | **6/30 (20.0%)** | **3/8 (37.5%)** | 14/43 (32.6%) | 7/18 (38.9%) | 3/7 (42.9%) | 4/13 (30.8%) |  | 20/55 (36.4%) |
| Duration >= 10 days | **2/28 (7.1%)** | **9/30 (30.0%)** | **2/8 (25.0%)** | 9/43 (20.9%) | 3/18 (16.7%) | 1/7 (14.3%) | 2/13 (15.4%) |  | 11/55 (20.0%) |
|  |  |  |  |  |  |  |  |  |  |
| De-escalation of antibiotics in specific situations | NW | SE | Asia | Adults | Children | Both | Auto | Allo | Both |
|  |  |  |  |  |  |  |  |  |  |
| Positive blood culture with susceptible pathogen with uncomplicated presentation | 62/83 (74.7%) | 66/82 (80.5%) | 12/18 (66.7%) | 93/122 (76.2%) | 26/35 (74.3%) | 24/29 (82.8%) | 31/40 (77.5%) | 3/3 (100%) | 109/143 (76.2%) |
| Positive blood culture with susceptible pathogen with severe presentation, improved on empirical therapy | 38/82 (46.3%) | 37/81 (45.7%) | 6/18 (33.3%) | 55/119 (46.2%) | 16/34 (47.1%) | 11/30 (36.7%) | 17/39 (43.6%) | 2/3 (66.7%) | 63/141 (44.7%) |
| Clinically documented infection with uncomplicated presentation, afebrile on empirical therapy | 46/80 (57.5%) | 54/82 (65.9%) | 11/19 (57.9%) | 73/119 (61.3%) | 22/35 (62.9%) | 18/29 (62.1%) | 24/40 (60.0%) | 2/3 (66.7%) | 87/140 (62.1%) |
| Clinically documented infection with severe presentation, improved and afebrile on empirical therapy | 30/81 (37.0%) | 33/82 (40.2%) | 7/19 (36.8%) | 41/120 (34.2%) | 15/35 (42.9%) | 15/29 (51.7%) | 14/40 (35.0%) | 1/3 (33.3%) | 56/141 (39.7%) |
| Fever of unknown origin with uncomplicated presentation, afebrile on empirical therapy | 38/82 (46.3%) | 49/82 (59.8%) | 11/19 (57.9%) | **63/120 (52.5%)** | **25/35 (71.4%)** | **12/30 (40.0%)** | 20/40 (50.0%) | 1/3 (33.3%) | 79/142 (55.6%) |
| Fever of unknown origin with severe presentation, improved and afebrile on empirical therapy | 24/82 (29.3%) | 24/82 (29.3%) | 7/19 (36.8%) | **33/120 (27.5%)** | **17/35 (48.6%)** | **6/30 (20.0%)** | 8/40 (20.0%) | 1/3 (33.3%) | 47/142 (33.1%) |
|  |  |  |  |  |  |  |  |  |  |
| Stop before neutrophil recovery in specific situations | NW | SE | Asia | Adults | Children | Both | Auto | Allo | Both |
|  |  |  |  |  |  |  |  |  |  |
| Positive blood culture with susceptible pathogen with uncomplicated presentation | **39/82 (47.6%)** | **23/82 (28.0%)** | **6/19 (31.6%)** | 42/121 (34.7%) | 15/35 (42.9%) | 11/30 (36.7%) | 16/40 (40.0%) | 2/3 (66.7%) | 50/143 (35.0%) |
| Positive blood culture with susceptible pathogen with severe presentation, improved on empirical therapy | 22/82 (26.8%) | 12/82 (14.6%) | 3/19 (15.8%) | 21/121 (17.4%) | 10/35 (28.6%) | 6/30 (20.0%) | 9/40 (22.5%) | 0/3 (0%) | 28/143 (19.6%) |
| Clinically documented infection with uncomplicated presentation, afebrile on empirical therapy | **49/82 (59.8%)** | **23/82 (28.0%)** | **4/19 (21.1%)** | 46/121 (38.0%) | 17/35 (48.6%) | 13/30 (43.3%) | 18/40 (45.0%) | 1/3 (33.3%) | 57/143 (39.9%) |
| Clinically documented infection with severe presentation, improved and afebrile on empirical therapy | 22/81 (27.2%) | 13/82 (15.9%) | 4/19 (21.1%) | 23/121 (19.0%) | 9/35 (25.7%) | 7/29 (24.1%) | 8/40 (20.0%) | 0/3 (0%) | 31/142 (21.8%) |
| Fever of unknown origin with uncomplicated presentation, afebrile on empirical therapy | **50/80 (62.5%)** | **30/82 (36.6%)** | **11/19 (57.9%)** | 56/120 (46.7%) | 19/35 (54.3%) | 16/29 (55.2%) | 25/40 (62.5%) | 1/3 (33.3%) | 65/141 (46.1%) |
| Fever of unknown origin with severe presentation, improved and afebrile on empirical therapy | 23/81 (28.4%) | 15/82 (18.3%) | 2/18 (11.1%) | 21/120 (17.5%) | 11/35 (31.4%) | 8/29 (27.6%) | 9/40 (22.5%) | 0/3 (0%) | 31/141 (22.0%) |
| Probable/proven pulmonary aspergillosis with uncomplicated presentation, afebrile on antifungal therapy | 18/79 (22.8%) | 17/82 (20.7%) | 6/19 (31.6%) | 30/119 (25.2%) | 5/35 (14.3%) | 6/29 (18.8%) | 8/39 (20.5%) | 0/3 (0%) | 33/141 (23.4%) |
| Probable/proven pulmonary aspergillosis with severe presentation, improved and afebrile on antifungal therapy | 14/79 (17.7%) | 14/82 (17.1%) | 5/19 (26.3%) | 21/119 (17.6%) | 5/35 (14.3%) | 7/29 (24.1%) | 5/39 (12.8%) | 0/3 (0%) | 28/141 (19.9%) |
|  |  |  |  |  |  |  |  |  |  |
| How long is antibiotic therapy generally continued | NW | SE | Asia | Adults | Children | Both | Auto | Allo | Both |
|  |  |  |  |  |  |  |  |  |  |
| Positive blood culture |  |  |  |  |  |  |  |  |  |
| < 7 days | 0/79 (0%) | 2/81 (2.5%) | 0/19 (0%) | 1/120 (0.8%) | 1/34 (2.9%) | 0/28 (0%) | 0/39 (0%) | 0/3 (0%) | 2/140 (1.4%) |
| 7-10 days | 32/79 (40.5%) | 17/81 (21.0%) | 2/19 (10.5%) | 37/120 (30.8%) | 8/34 (23.5%) | 6/28 (21.4%) | 19/39 (48.7%) | 1/3 (33.3%) | 31/140 (22.1%) |
| 11-14 days | 21/79 (26.6%) | 28/81 (34.6%) | 13/19 (68.4%) | 40/120 (33.3%) | 15/34 (44.1%) | 8/28 (28.6%) | 8/39 (20.5%) | 1/3 (33.3%) | 54/140 (38.6%) |
| 15-21 days | 4/79 (5.1%) | 7/81 (8.6%) | 0/19 (0%) | 10/120 (8.3%) | 0/34 (0%) | 1/28 (3.6%) | 3/39 (7.7%) | 1/3 (33.3%) | 7/140 (5.0%) |
| until end of neutropenia | 22/79 (27.8%) | 27/81 (33.3%) | 4/19 (21.1%) | 32/120 (26.7%) | 10/34 (29.4%) | 13/28 (46.4%) | 9/39 (23.1%) | 0/3 (0%) | 46/140 (32.9%) |
| Clinically documented infection |  |  |  |  |  |  |  |  |  |
| < 7 days | 3/79 (3.8%) | 1/81 (1.2%) | 0/19 (0%) | 2/120 (1.7%) | 1/34 (2.9%) | 1/28 (3.6%) | 0/39 (0%) | 0/3 (0%) | 4/140 (2.9%) |
| 7-10 days | 33/79 (41.8%) | 17/81 (21.0%) | 7/19 (36.8%) | 44/120 (36.7%) | 9/34 (26.5%) | 4/28 (14.3%) | 20/39 (51.3%) | 1/3 (33.3%) | 36/140 (25.7%) |
| 11-14 days | 21/79 (26.6%) | 34/81 (42.0%) | 8/19 (42.1%) | 37/120 (30.8%) | 16/34 (47.1%) | 10/28 (35.7%) | 11/39 (28.2%) | 2/3 (66.7%) | 50/140 (35.7%) |
| 15-21 days | 2/79 (2.5%) | 4/81 (4.9%) | 1/19 (5.3%) | 6/120 (5.0%) | 0/34 (0%) | 1/28 (3.6%) | 1/39 (2.6%) | 0/3 (0%) | 6/140 (6.3%) |
| until end of neutropenia | 20/79 (25.3%) | 25/81 (30.9%) | 3/19 (15.8%) | 31/120 (25.8%) | 8/34 (23.5%) | 12/28 (42.9%) | 7/39 (17.9%) | 0/3 (0%) | 44/140 (41.3%) |
| Fever of unknown origin |  |  |  |  |  |  |  |  |  |
| < 7 days | 21/79 (26.6%) | 9/81 (10.8%) | 2/19 (10.5%) | 21/120 (17.5%) | 9/34 (26.5%) | 2/28 (7.1%) | 8/39 (20.5%) | 0/3 (0%) | 24/140 (17.1%) |
| 7-10 days | 26/79 (32.9%) | 28/81 (34.6%) | 6/19 (31.6%) | 41/120 (34.2%) | 12/34 (35.3%) | 7/28 (25.0%) | 17/39 (43.6%) | 1/3 (33.3%) | 42/140 (30.0%) |
| 11-14 days | 7/79 (8.9%) | 17/81 (21.0%) | 3/19 (15.8%) | 17/120 (14.2%) | 6/34 (17.6%) | 4/28 (14.3%) | 5/39 (12.8%) | 0/3 (0%) | 22/140 (15.7%) |
| 15-21 days | 0/79 (0%) | 2/81 (2.5%) | 1/19 (5.3%) | 2/120 (1.7%) | 0/34 (0%) | 1/28 (3.6%) | 1/39 (2.6%) | 0/3 (0%) | 2/140 (1.4%) |
| until end of neutropenia | 25/79 (31.6%) | 25/81 (30.9%) | 7/19 (36.8%) | 39/120 (32.5%) | 7/34 (20.6%) | 14/28 (50.0%) | 8/39 (20.5%) | 2/3 (66.7%) | 50/140 (35.7%) |

Supplementary Table 1: Comparison by geographical region, type of transplant and patient age

This table summarizes comparison of responses by geographical region, type of transplant and patient age.

For further analyses on geographical variation in antibiotic policies, the centers were divided in three geographical zones: north/west (NW), including Austria, Belgium, Denmark, Finland, France, Germany, Ireland, the Netherlands, Sweden, Switzerland and the UK; south/east (SE), including Bulgaria, the Czech Republic, Estonia, Greece, Hungary, Israel, Italy, Lithuania, Poland, Portugal, Romania, Russia, Slovakia and Spain; Asia, including Cyprus, India, Iran, Israel, Jordan, Pakistan, Qatar and Turkey.
