# Supplementary material for: Current antimicrobial practice in febrile neutropenia across Europe and Asia: the EBMT Infectious Disease Working Party survey
Source: Bone Marrow Transplant. 2020 Feb 11;55(8):1588–94. doi: 10.1038/s41409-020-0811-y (PMC7391284; doi:10.1038/s41409-020-0811-y)
Supplement: Supplementary file 2 — Supplementary Table 2: Implementation of de-escalation/discontinuation strategies relative to other variables [file 41409_2020_811_MOESM2_ESM.docx]

| Implementation of de-escalation/discontinuation strategies |  |  |  |  |  |  |  |  |  |  |  |  |
| --- | --- | --- | --- | --- | --- | --- | --- | --- | --- | --- | --- | --- |
|  | IDM guide + | IDM guide - | IDM dec + | IDM dec - | FP + | FP - | Combo + | Combo - | Glycopep + | Glycopep - | Escalate + | Escalate - |
| Empirical de-escalation of combination therapy | n = 111 | n = 71 | n = 98 | n = 95 | n = 109 | n = 82 | n = 71 | n = 119 | n = 115 | n = 74 | n = 135 | n = 54 |
|  |  |  |  |  |  |  |  |  |  |  |  |  |
| Combination therapy empirically in first line in stable patients without history of resistant pathogens | 37/109 (33.9%) | 30/69 (43.5%) | 33/97 (34.0%) | 38/92 (41.3%) | 41/107 (38.3%) | 30/82 (36.6%) |  |  | 47/115 (40.9%) | 24/74 (32.4%) | 52/135 (38.5%) | 19/54 (35.2%) |
| Duration =< 3 days | 16/36 (44.4%) | 7/29 (24.1%) | 14/31 (45.2%) | 10/37 (27.0%) | 11/40  (27.5%) | 13/28 (46.4%) |  |  | 14/46 (30.4%) | 10/22 (45.5%) | **12/49 (24.5%)** | **12/19 (63.2%)** |
| Duration >= 10 days | 5/36 (13.9%) | 6/29 (20.7%) | 3/31 (9.7%) | 10/37 (27.0%) | 10/40 (25.0%) | 3/28 (3.7%) |  |  | 9/46 (19.6%) | 4/22 (18.2%) | **13/49 (26.5%)** | **0/19 (0%)** |
|  |  |  |  |  |  |  |  |  |  |  |  |  |
| De-escalation of antibiotics in specific situations | IDM guide + | IDM guide - | IDM  dec + | IDM dec - | FQ+ | FQ- | Combo+ | Combo- | Glycopep+ | Glycopep- | Escalate+ | Escalate- |
|  |  |  |  |  |  |  |  |  |  |  |  |  |
| Positive blood culture with susceptible pathogen with uncomplicated presentation | 81/108 (75.0%) | 51/66 (77.3%) | 75/96 (78.1%) | 68/89 (76.4%) | 86/107 (80.4%) | 56/78 (71.8%) | 52/69 (75.3%) | 91/117 (77.8%) | 85/112 (75.9%) | 57/73 (78.1%) | 98/133 (73.7%) | 44/52 (84.6%) |
| Positive blood culture with susceptible pathogen with severe presentation, improved on empirical therapy | 53/108 (49.1%) | 25/66 (37.9%) | **50/94 (53.2%)** | **32/88 (36.4%)** | 47/104 (45.2%) | 34/78 (43.6%) | 29/66 (43.9%) | 53/117 (45.3%) | 49/111 (44.1%) | 32/71 (45.1%) | 56/131 (42.7%) | 26/51 (51.0%) |
| Clinically documented infection with uncomplicated presentation, afebrile on empirical therapy | 68/105 (64.8%) | 37/66 (56.1%) | 58/93 (62.4%) | 55/89 (61.8%) | 65/104 (62.5%) | 48/78 (61.5%) | 41/68 (60.3%) | 72/115 (62.6%) | 70/112 (62.5%) | 42/70 (60.0%) | 77/132 (58.3%) | 36/50 (72.0%) |
| Clinically documented infection with severe presentation, improved and afebrile on empirical therapy | 41/106 (38.7%) | 25/66 (37.9%) | 42/93 (45.2%) | 29/90 (32.2%) | 40/105 (38.1%) | 31/78 (39.7%) | 25/68 (36.8%) | 46/116 (39.7%) | 45/112 (40.2%) | 25/71 (35.2%) | 48/132 (36.4%) | 23/51 (45.1%) |
| Fever of unknown origin with uncomplicated presentation, afebrile on empirical therapy | 54/107 (50.5%) | 39/66 (59.1%) | 53/94 (56.4%) | 47/90 (52.2%) | 57/106 (53.8%) | 43/78 (55.1%) | **44/68 (64.7%)** | **56/117 (47.9%)** | 58/112 (51.8%) | 41/72 (56.9%) | 68/133 (51.1%) | 32/51 (62.7%) |
| Fever of unknown origin with severe presentation, improved and afebrile on empirical therapy | 36/107 (33.6%) | 18/66 (27.3%) | **36/94 (38.3%)** | **20/90 (22.2%)** | 31/106 (29.2%) | 25/78 (32.1%) | 22/68 (32.4%) | 34/117 (29.1%) | 35/112 (31.3%) | 20/72 (27.8%) | 37/133 (27.8%) | 19/51 (37.3%) |
|  |  |  |  |  |  |  |  |  |  |  |  |  |
| Stop before neutrophil recovery in specific situations | IDM guide + | IDM guide - | IDM dec + | IDM dec - | FQ+ | FQ- | Combo+ | Combo- | Glycopep+ | Glycopep- | Escalate+ | Escalate- |
|  |  |  |  |  |  |  |  |  |  |  |  |  |
| Positive blood culture with susceptible pathogen with uncomplicated presentation | **48/107 (44.9%)** | **18/67 (26.9%)** | 40/95 (42.1%) | 28/90 (31.1%) | 40/107 (37.4%) | 28/78 (35.9%) | 22/68 (32.4%) | 46/118 (39.0%) | 37/112 (33.0%) | 31/73 (42.5%) | **41/133 (30.8%)** | **27/52 (51.9%)** |
| Positive blood culture with susceptible pathogen with severe presentation, improved on empirical therapy | **29/107 (27.1%)** | **8/67 (11.9%)** | 23/95 (24.2%) | 14/90 (15.6%) | 23/107 (21.5%) | 14/78 (17.9%) | 15/68 (22.1%) | 22/118 (18.6%) | 20/112 (17.9%) | 17/73 (23.3%) | 25/133 (18.8%) | 12/52 (23.1%) |
| Clinically documented infection with uncomplicated presentation, afebrile on empirical therapy | **52/107 (48.6%)** | **22/67 (32.8%)** | 43/95 (45.3%) | 33/90 (36.7%) | 43/107 (40.2%) | 33/78 (42.3%) | 26/68 (38.2%) | 50/118 (42.4%) | 42/112 (37.5%) | 34/73 (46.6%) | 52/133 (39.1%) | 24/52 (46.2%) |
| Clinically documented infection with severe presentation, improved and afebrile on empirical therapy | 27/106 (25.5%) | 11/67 (16.4%) | 24/94 (25.5%) | 15/90 (16.7%) | 24/107 (22.4%) | 15/77 (19.5%) | 12/68 (17.6%) | 27/117 (23.1%) | 20/112 (17.9%) | 19/72 (26.4%) | 25/132 (18.9%) | 14/52 (26.9%) |
| Fever of unknown origin with uncomplicated presentation, afebrile on empirical therapy | 58/106 (54.7%) | 29/66 (43.9%) | 53/94 (56.4%) | 38/89 (42.7%) | 52/106 (49.1%) | 39/77 (50.6%) | 31/67 (46.3%) | 60/117 (51.3%) | **48/111 (43.2%)** | **43/72 (59.7%)** | 60/131 (45.8%) | 31/52 (59.6%) |
| Fever of unknown origin with severe presentation, improved and afebrile on empirical therapy | **32/106 (30.2%)** | **8/66 (12.1%)** | 24/93 (25.8%) | 16/90 (17.8%) | 24/106 (22.6%) | 16/77 (20.8%) | 14/68 (20.6%) | 26/116 (22.4%) | 20/111 (18.0%) | 20/72 (27.8%) | 27/131 (20.6%) | 13/52 (25.0%) |
| Probable/proven pulmonary aspergillosis with uncomplicated presentation, afebrile on antifungal therapy | 23/105 (21.9%) | 15/66 (22.7%) | 24/93 (25.8%) | 17/89 (19.1%) | 25/106 (23.6%) | 16/76 (21.1%) | 13/66 (19.7%) | 28/117 (23.9%) | **19/111 (17.1%)** | **21/71 (29.6%)** | 28/130 (21.5%) | 13/52 (25.0%) |
| Probable/proven pulmonary aspergillosis with severe presentation, improved and afebrile on antifungal therapy | 20/105 (19.0%) | 11/67 (16.4%) | 20/92 (21.7%) | 13/90 (14.4%) | 21/106 (19.8%) | 12/76 (15.8%) | 11/67 (16.4%) | 22/116 (19.0%) | 15/112 (13.4%) | 17/70 (24.3%) | 22/131 (16.8%) | 11/51 (21.6%) |
|  |  |  |  |  |  |  |  |  |  |  |  |  |

| How long is antibiotic therapy generally continued | IDM guide + | IDM guide - | IDM dec + | IDM dec - | FQ+ | FQ- | Combo+ | Combo- | Glycopep+ | Glycopep- | Escalate+ | Escalate- |
| --- | --- | --- | --- | --- | --- | --- | --- | --- | --- | --- | --- | --- |
|  |  |  |  |  |  |  |  |  |  |  |  |  |
| Positive blood culture |  |  |  |  |  |  |  |  |  |  |  |  |
| < 7 days | 1/104 (1.0%) | 1/66 (1.5%) | 1/94 (1.1%) | 1/87 (1.1%) | 2/106 (1.9%) | 0/75 (0.0%) | 0/68 (0%) | 2/114 (1.8%) | 2/111 (1.8%) | 0/70 (0%) | 1/129 (0.8%) | 1/52 (1.9%) |
| 7-10 days | 37/104 (35.6%) | 14/66 (21.2%) | 32/94 (34.0%) | 19/87 (21.8%) | 29/106 (27.4%) | 22/75 (29.3%) | 20/68 (29.4%) | 31/114 (27.2%) | 30/111 (27.0%) | 21/70 (30.0%) | 36/129 (27.9%) | 15/52 (28.8%) |
| 11-14 days | 33/104 (31.7%) | 22/66 (33.3%) | 34/94 (36.2%) | 29/87 (33.3%) | 37/106 (34.9%) | 25/75 (33.3%) | 21/68 (30.9%) | 42/114 (36.8%) | 37/111 (33.3%) | 25/70 (35.7%) | 40/129 (31.0%) | 22/52 (42.3%) |
| 15-21 days | 5/104 (4.8%) | 6/66 (9.1%) | 5/94 (5.3%) | 5/87 (5.7%) | 6/106 (5.7%) | 5/75 (6.7%) | 2/68 (2.9%) | 9/119 (7.9%) | 4/111 (3.6%) | 7/70 (10.0%) | 9/129 (7.0%) | 2/52 (3.8%) |
| until end of neutropenia | 28/104 (26.9%) | 23/66 (34.8%) | **22/94 (23.4%)** | **33/87 (37.9%)** | 32/106 (30.2%) | 23/75 (30.7%) | 25/68 (36.8%) | 30/114 (26.3%) | 38/111 (34.2%) | 17/70 (24.3%) | 43/129 (33.3%) | 12/52 (23.1%) |
| Clinically documented infection |  |  |  |  |  |  |  |  |  |  |  |  |
| < 7 days | 3/104 (2.9%) | 0/66 (0%) | 2/94 (2.1%) | 2/87 (2.3%) | 3/106 (2.8%) | 1/75 (1.3%) | 1/68 (1.5%) | 3/114 (2.6%) | 2/111 (1.8%) | 2/70 (2.9%) | 2/129 (1.6%) | 2/52 (3.8%) |
| 7-10 days | 36/104 (34.6%) | 17/66 (25.8%) | 40/94 (42.6%) | 17/87 (19.5%) | 32/106 (30.2%) | 25/75 (33.3%) | 25/68 (36.8%) | 32/114 (28.1%) | 32/111 (28.8%) | 25/70 (35.7%) | 37/129 (28.7%) | 19/52 (36.5%) |
| 11-14 days | 37/104 (35.6%) | 23/66 (34.8%) | 29/94 (30.9%) | 33/87 (37.9%) | 36/106 (34.0%) | 26/75 (34.7%) | 17/68 (25.0%) | 46/114 (40.4%) | 38/111 (34.2%) | 24/70 (34.3%) | 45/129 (34.9%) | 18/52 (34.6%) |
| 15-21 days | 3/104 (2.9%) | 4/66 (6.1%) | 3/94 (3.2%) | 4/87 (4.6%) | 4/106 (3.8%) | 3/75 (4.0%) | 2/68 (2.9%) | 5/114 (4.4%) | 4/111 (3.6%) | 3/70 (4.3%) | 6/129 (4.7%) | 1/52  (1.9%) |
| until end of neutropenia | 25/104 (24.0%) | 22/66 (33.3%) | **20/94 (21.3%)** | **31/87 (35.6%)** | 31/106 (29.2%) | 20/75 (26.7%) | 23/68 (33.8%) | 28/114 (24.6%) | 35/111 (31.5%) | 16/70 (22.9%) | 39/129 (30.2%) | 12/52 (23.1%) |
| Fever of unknown origin |  |  |  |  |  |  |  |  |  |  |  |  |
| < 7 days | 19/104 (18.3%) | 12/66 (18.2%) | 18/94 (19.1%) | 14/87 (16.1%) | 21/106 (19.8%) | 11/75 (14.7%) | 16/68 (23.5%) | 16/114 (14.0%) | 17/111 (15.3%) | 15/70 (21.4%) | 19/129 (14.7%) | 13/52 (25.0%) |
| 7-10 days | 40/104 (38.5%) | 17/66 (25.8%) | 33/94 (35.1%) | 26/87 (29.9%) | 31/106 (29.2%) | 29/75 (38.7%) | 22/68 (32.4%) | 38/114 (33.3%) | 37/111 (33.3%) | 23/70 (32.9%) | 43/129 (33.3%) | 17/52 (32.7%) |
| 11-14 days | 13/104 (12.5%) | 12/66 (18.2%) | 16/94 (17.0%) | 11/87 (12.6%) | 19/106 (17.9%) | 8/75 (10.7%) | 8/68 (11.8%) | 19/114 (16.7%) | 15/111 (13.5%) | 11/70 (15.7%) | 23/129 (17.8%) | 4/52 (7.7%) |
| 15-21 days | 2/104 (1.9%) | 1/66 (1.5%) | 3/94 (3.2%) | 0/87 (0%) | 2/106 (1.9%) | 0/75 (0.0%) | 1/68 (1.5%) | 2/114 (1.8%) | 2/111 (1.8%) | 1/70 (1.4%) | 2/129 (1.6%) | 1/52 (1.9%) |
| until end of neutropenia | 30/104 (28.8%) | 24/66 (36.4%) | **24/94 (25.5%)** | **36/87 (41.4%)** | 33/106 (31.1%) | 27/75 (36.0%) | 21/68 (30.9%) | 39/114 (34.2%) | 40/111 (36.0%) | 20/70 (28.6%) | 42/129 (32.6%) | 17/52 (32.7%) |

Supplementary Table 2: Implementation of de-escalation/discontinuation strategies relative to other variables

This table summarizes responses from centers on questions concerning Implementation of de-escalation/discontinuation strategies, compared by other variables.

IDM guide: Infectious disease or microbiology departments are involved in writing guidelines

IDM dec: Infectious disease or microbiology departments are involved in decision making on antimicrobial treatment

FP: fluoroquinolone prophylaxis

Combo: empirical combination therapy as first-line in stable patients without history of colonization/infection with resistant bacteria

Glycopep: empirical addition of a glycopeptide when fever persists for more than 2-3 days

Escalate: empirical escalation to a broader spectrum agent is performed when fever persists for more than 3-5 days
