# Supplementary material for: Current antimicrobial practice in febrile neutropenia across Europe and Asia: the EBMT Infectious Disease Working Party survey
Source: Bone Marrow Transplant. 2020 Feb 11;55(8):1588–94. doi: 10.1038/s41409-020-0811-y (PMC7391284; doi:10.1038/s41409-020-0811-y)
Supplement: Supplementary file 3 — Survey [file 41409_2020_811_MOESM3_ESM.docx]

| **Antibiotic use in febrile neutropenia in HSCT recipients**  EBMT/IDWP Survey |
| --- |
|  |

Centre information:

EBMT Centre Identification Code (CIC) : .......... .......... ..........

Hospital: ............................................................................................................... **U**

City: ............................................................................................................... **U**

Country: ............................................................................................................... **U**

Form completed by (name): ............................................................................................................... **U**

E-mail address: ...............................................................................................................

I agree to participate in this survey:

**🞎** Yes

**🞎** No, reason: ……………………………………………………………………………………..

**RHESPAT**

| Survey |
| --- |

**1. Your centre performs HSCT in::**

**🞎** Adults

**🞎** Children

**🞎** Both adults and children

**2. Your centre performs:**

**🞎** Autologous HSCT

**🞎** Allogeneic HSCT

**🞎** Both

**3. Are there institutional or departmental guidelines/instructions dealing with antibiotic policy?**

**🞎** No

**🞎** Yes, written by the Hematological department exclusively

**🞎** Yes, written by the Hematological department in cooperation with ……………………………………………………….. …….............................................................................................................................................................................................................................................................................................................

**4. Decisions on antimicrobial treatment on your transplant ward are primarily made by**

**🞎** Hematological department exclusively

**🞎** Hematological department in cooperation with ……………………………………………………………………………….

…….............................................................................................................................................................................................................................................................................................................

**5. Are there institutional restrictions on the use of specific antibiotics or the duration of antimicrobial treatment in your centre?**

**🞎** No

**🞎** Yes (please specify) …………………………………………………………………………………………………………….. …………………………………………………………………………………………………………………………………………………………………………………………………………………………………………………………………………………….

**6. Is your service regularly updated on (changes in) epidemiology, including prevalence of specific resistant bacteria and trends in resistance?**

**🞎** No 🡪 *Please continue to question 8*

**🞎** Yes

**7. Frequency of updates**

**🞎** Every 3 months

**🞎** Every 6 months

**🞎** Every 12 months

**🞎** Every 24 months

**🞎** Every 36 months

**🞎** Other (please specify)……………………………………………………………………………………………………………

**8. Are surveillance cultures performed at your centre?**

**🞎** No 🡪 *Please continue to question 11*

**🞎** Yes

**9. Surveillance cultures are performed in which patients?**

**🞎** Autologous

**🞎** Allogeneic

**🞎** Both Autologous and Allogeneic

**🞎** Other (please specify)………………………………………………………………………………………………………….

………………………………………………………………………………………………………………………………………..

**10. On which sites and how often are surveillance cultures usually performed?**

|  | **Weekly** | **2x per week** | **Biweekly** | **Monthly** | **Only on admission** | **Never** | **Other** |
| --- | --- | --- | --- | --- | --- | --- | --- |
| **Nose Swab** |  |  |  |  |  |  |  |
| **Throat swab** |  |  |  |  |  |  |  |
| **Urine sample** |  |  |  |  |  |  |  |
| **Stool sample** |  |  |  |  |  |  |  |
| **Central line swab** |  |  |  |  |  |  |  |
| **Blood cultures** |  |  |  |  |  |  |  |
| **Rectal swab** |  |  |  |  |  |  |  |

**11. Is there rapid (within 24 hours after blood culture positivity) reporting of positive blood cultures at your centre?**

**🞎** No

**🞎** Yes

**12. Is there active (e.g. by telephone) reporting of positive blood cultures at your centre?**

**🞎** No

**🞎** Yes

**13. Is the resistance pattern of positive blood cultures reported within 24 hours after it becomes positive?**

**🞎** No

**🞎** Yes

**14. Is fluoroquinolone prophylaxis generally used at your centre?**

**🞎** Yes

**🞎** No, never used 🡪*Please continue to question 22*

**🞎** No, stopped (please specify when and why) ………………………………………………………………………………..

…………………………………………………………………………………………………………………………………………

………………………………………………………………………………………………………………………………………………………………………………………………………………………………………… *🡪 Please continue to question 22*

**15. Fluoroquinolone prophylaxis is used in:**

|  | **Adults** | **Children** | **Both** |
| --- | --- | --- | --- |
| **Autologous HSCT** |  |  |  |
| **Allogeneic HSCT** |  |  |  |

**16. Which type of Fluoroquinolone prophylaxis is used?**

**Autologous**

**🞎** Ciprofloxacine

**🞎** Levofloxacine

**🞎** Norfloxacine

**🞎** Ofloxacine

**🞎** Other:…………………………………………………………………………………………………………………………

**Allogeneic**

**🞎** Ciprofloxacine

**🞎** Levofloxacine

**🞎** Norfloxacine

**🞎** Ofloxacine

**🞎** Other:…………………………………………………………………………………………………………………………

**17. What dose in mg/day of Fluoroquinolone prophylaxis is used?**

……………………………………………………………………………….

**18. Fluoroquinolone prophylaxis is started**

**🞎** At onset of chemotherapy

**🞎** At onset of neutropenia

**🞎** Other:………………………………………………………………………………………………………………………….

**19. Fluoroquinolone prophylaxis is started at onset of neutropenia**

**(ANC < ………………x 10^9^/L)**

**20. Fluoroquinolone prophylaxis is continued until**

**🞎** Discharge of the patient

**🞎** Recovery of neutrophils

**🞎** Other (please specify) ..................................................................................................................................................

………………………………………………………………………………………………………………………………………..

**21. Fluoroquinolone prophylaxis is continued until recovery of neutrophils**

**(ANC > …………….x 10^9^/L)**

**22. Is co-trimoxazole prophylaxis generally used at your centre?**

**🞎** No *🡪 Please continue to question 30*

**🞎** Yes

**23. Co-trimoxazole prophylaxis is used in**

|  | **Adults** | **Children** | **Both** |
| --- | --- | --- | --- |
| **Autologous HSCT** |  |  |  |
| **Allogeneic HSCT** |  |  |  |

**24. What total dose per week co-trimoxazole prophylaxis is generally used at your centre?**

......................................................................................................................................................................................

**25. Co-trimoxazole prophylaxis is started**

**🞎** At the onset of chemotherapy

**🞎** At onset of neutropenia
**🞎** After engraftment
**🞎** Other (please specify) ………………………………………………………………………………………………………..

**26. Co-trimoxazole prophylaxis is started at onset of neutropenia**

**(ANC < ……………x 10^9^/L)**

**27. Co-trimoxazole prophylaxis is continued until ....... months post-autologous transplantation**

**28. Co-trimoxazole prophylaxis is continued until ....... months post-allogeneic transplantation**

**29. Co-trimoxazole prophylaxis is continued until; other** (please specify)…………………….

………………………………………………………………………………………………………………

**30. When considering all episodes of febrile neutropenia, how frequently is a de-escalation strategy used versus an escalation strategy?** (sum should be 100%)

De-Escalation strategy used in ………….%

Escalation strategy used in ………….%

**31. What first line empiric antimicrobial therapy is generally used for febrile neutropenia at your centre?** (If two therapies are used equally, please specify)

**🞎** Cefepime

**🞎** Carbapenem
**🞎** Piperacilline/tazobactam
**🞎** Other (please specify)…………………………………………………………………………………………………………..

**32. Does your centre generally use combination therapy empirically in first line in stable patients without history of colonization/infection with resistant pathogens?**

**🞎** No *🡪 Please continue to question 35*

**🞎** Yes

**33. What combination therapy does your centre generally use empirically in first line in stable patients without history of colonization/infection with resistant pathogens. Products** (please specify)**:** ………………………………………………………………………………

………………………………………………………………………………………………………………………………………………………………………………………………………………………………

**34. Continued for how many days?**

………………………………………….

**35. Is association of a glycopeptide for gram+ coverage performed empirically in case of persistent fever?**

**🞎** No

**🞎** Yes, after ….. days of fever *🡪 Please continue to question 37*

**36. Only in case of** (multiple responses possible)

**🞎** Septic shock

**🞎** Clinical suspicion of gram+ infection
**🞎** Microbiological cultures showing resistant pathogen
**🞎** Other (please specify) …………………………………………………………………………………………………………

**37. Is escalation to a broader spectrum agent (e.g. carbapenem) performed empirically in case of persistent fever?**

**🞎** No

**🞎** Yes, after ….. days of fever *🡪 Please continue to question 39*

**38. Only in case of (multiple responses possible)**

**🞎** Septic shock
**🞎** Microbiological cultures showing resistant pathogen
**🞎** Other (please specify) …………………………………………………………………………………………………………

*Do you de-escalate to a narrower spectrum agent in the following scenario’s and if so, how often?*

**39. Positive blood cultures with a susceptible pathogen and uncomplicated presentation**

**🞎** No

**🞎** Yes, in ….. % of such situations

**40. Positive blood cultures with a susceptible pathogen and severe presentation which improved on empirical therapy**

**🞎** No

**🞎** Yes, in ….. % of such situations

**41. Clinically documented infection with uncomplicated presentation, afebrile on empirical therapy**

**🞎** No

**🞎** Yes, in ….. % of such situations

**42. Clinically documented infection with severe presentation, but improved on empirical therapy and became afebrile**

**🞎** No

**🞎** Yes, in ….. % of such situations

**43. Fever of unknown origin with uncomplicated presentation, afebrile on empirical therapy**

**🞎** No

**🞎** Yes, in ….. % of such situations

**44. Fever of unknown origin with severe presentation, but improved on empirical therapy and became afebrile**

**🞎** No

**🞎** Yes, in ….. % of such situations

**45. Do you stop antimicrobial therapy before neutrophil recovery in positive blood cultures with a susceptible pathogen and uncomplicated presentation**

**🞎** No

**🞎** Yes, in ….. % of such situations / after …… days without fever

**46. Do you stop antimicrobial therapy before neutrophil recovery in positive blood cultures with a susceptible pathogen and severe presentation which improved on empirical therapy?**

**🞎** No

**🞎** Yes, in ….. % of such situations / after …… days without fever

**47. Do you stop antimicrobial therapy before neutrophil recovery in a clinically documented infection with uncomplicated presentation, afebrile on empirical therapy?**

**🞎** No

**🞎** Yes, in ….. % of such situations / after …… days without fever

**48. Do you stop antimicrobial therapy before neutrophil recovery in a clinically documented infection with severe presentation, but improved on empirical therapy and became afebrile?**

**🞎** No

**🞎** Yes, in ….. % of such situations / after …… days without fever

**49. Do you stop antimicrobial therapy before neutrophil recovery in case of probable/proven invasive pulmonary aspergillosis, with uncomplicated presentation, afebrile on antifungal therapy?**

**🞎** No

**🞎** Yes, in ….. % of such situations / after …… days without fever

**50. Do you stop antimicrobial therapy before neutrophil recovery in case of probable/proven invasive pulmonary aspergillosis, with severe presentation, but improved on antifungal therapy and became afebrile?**

**🞎** No

**🞎** Yes, in ….. % of such situations / after …… days without fever

**51. Do you stop antimicrobial therapy before neutrophil recovery in case of fever of unknown origin with uncomplicated presentation, afebrile on empirical therapy?**

**🞎** No

**🞎** Yes, in ….. % of such situations / after …… days without fever

**52. Do you stop antimicrobial therapy before neutrophil recovery in case of fever of unknown origin with severe presentation, but improved on empirical therapy and became afebrile?**

**🞎** No

**🞎** Yes, in ….. % of such situations / after …… days without fever

**53. How long is antibiotic treatment generally continued?**

**Positive blood culture**

**🞎** <7 days

**🞎** 7-10 days
**🞎** 11-14 days **🞎** 15-21 days
**🞎** until end of neutropenia

**Clinically documented infection**

**🞎** <7 days

**🞎** 7-10 days
**🞎** 11-14 days **🞎** 15-21 days
**🞎** until end of neutropenia

**Fever of unknown origin**

**🞎** <7 days

**🞎** 7-10 days
**🞎** 11-14 days **🞎** 15-21 days
**🞎** until end of neutropenia

**54. How frequently are resistant pathogens isolated from cultures at your centre (over the past 12 months)?**

|  | Surveillance in % | Blood culture in % | Not done |
| --- | --- | --- | --- |
| Pathogen: Staph Aureus,  Type of resistance: MRSA |  |  |  |
| Pathogen: CNS,  Type of resistance: MR |  |  |  |
| Pathogen: Enterococci,  Type of resistance: VRE |  |  |  |
| Pathogen: Gram -,  Type of resistance: FQ resistant |  |  |  |
| Pathogen: Gram -,  Type of resistance: 3rd gen cephalosporin-resistant |  |  |  |
| Pathogen: Gram -,  Type of resistance: Carbapenem |  |  |  |
| Pathogen: Enterobacteriaceae, Type of resistance: ESBL-producers |  |  |  |
| Pathogen: P. aeruginosa,  Type of resistance: Carbapenem-resistant |  |  |  |

**Comments**

***Thank you!***

***Please send the completed form to:***

(you may use e-mail, fax, or post)

**EBMT Data Office Leiden / IDWP**Department of Medical Statistics & Bioinformatics

S-05-P, Leiden University Medical Center

PO Box 9600

2300 RC, Leiden, The Netherlands

Fax +49 711 4900 8723 / +49 180 500 290 623

E-mail:  [idwpebmt@lumc.nl](mailto:idwpebmt@lumc.nl)
